# Machine learning with routine electronic medical record data to identify people at high risk of disengagement from HIV care in Tanzania

**DOI:** 10.1371/journal.pgph.0000720

**Published:** 2022-09-16

**Authors:** Carolyn A. Fahey, Linqing Wei, Prosper F. Njau, Siraji Shabani, Sylvester Kwilasa, Werner Maokola, Laura Packel, Zeyu Zheng, Jingshen Wang, Sandra I. McCoy

**Affiliations:** 1 Department of Epidemiology, School of Public Health, University of Washington, Seattle, Washington, United States of America; 2 Division of Biostatistics, School of Public Health, University of California, Berkeley, California, United States of America; 3 Ministry of Health, Dodoma, Tanzania; 4 Division of Epidemiology, School of Public Health, University of California, Berkeley, California, United States of America; 5 Department of Industrial Engineering and Operations Research, University of California, Berkeley, California, United States of America; Duke University, UNITED STATES

## Abstract

Machine learning methods for health care delivery optimization have the potential to improve retention in HIV care, a critical target of global efforts to end the epidemic. However, these methods have not been widely applied to medical record data in low- and middle-income countries. We used an ensemble decision tree approach to predict risk of disengagement from HIV care (missing an appointment by ≥28 days) in Tanzania. Our approach used routine electronic medical records (EMR) from the time of antiretroviral therapy (ART) initiation through 24 months of follow-up for 178 adults (63% female). We compared prediction accuracy when using EMR-based predictors alone and in combination with sociodemographic survey data collected by a research study. Models that included only EMR-based indicators and incorporated changes across past clinical visits achieved a mean accuracy of 75.2% for predicting risk of disengagement in the next 6 months, with a mean sensitivity of 54.7% for targeting the 30% highest-risk individuals. Additionally including survey-based predictors only modestly improved model performance. The most important variables for prediction were time-varying EMR indicators including changes in treatment status, body weight, and WHO clinical stage. Machine learning methods applied to existing EMR data in resource-constrained settings can predict individuals’ future risk of disengagement from HIV care, potentially enabling better targeting and efficiency of interventions to promote retention in care.

## Introduction

Forty years into the HIV epidemic, disengagement from care and poor antiretroviral therapy (ART) adherence remain central challenges that undermine global efforts for epidemic control. Only 65% of people living with HIV (PLHIV) in eastern and southern Africa have viral suppression, a partial reflection of persistent attrition from care and suboptimal adherence to ART [1]. Especially in sub-Saharan Africa, lifelong retention in HIV care is continually threatened by myriad barriers such as stigma, food insecurity, negative clinic experiences, anticipated or actual side effects, misinformation, “treatment fatigue,” and poverty [2]. Consequently, retention is a dynamic process, as PLHIV may default and re-engage in care numerous times over a lifetime, such that 33% of PLHIV starting ART in sub-Saharan Africa between 2009 and 2014 were not alive and/or on ART after five years [3].

Fortunately, there is a burgeoning literature on effective strategies to bolster engagement with HIV care. This includes, for example: enhanced adherence counseling in response to high viremia, an approach common across sub-Saharan Africa [4]; reminder text messages, an inexpensive and highly scalable approach [5]; and short-term financial incentives, an approach that can support habit formation and nudge PLHIV towards HIV care [6,7]. Nevertheless, effect sizes of such interventions are often modest, especially after intervention periods are complete and effectiveness gradually wanes [8,9]. Furthermore, even in well-conducted trials which find that an intervention has significant benefits, a sizable proportion of participants in the comparison group achieve the desired outcomes (e.g., viral suppression) *without* the intervention. Thus, scale-up of behavioral programs to improve adherence and retention may be difficult to justify, especially in resource-constrained environments.

An alternative approach is to better target interventions to the subset of the population that is most in need, rather than ‘one size fits all’ approaches that can be costly at scale. A practical challenge of better targeting is that until recently, customized behavioral interventions would have been cumbersome to seamlessly integrate into busy healthcare settings. However, the growth of electronic medical record (EMR) data in sub-Saharan Africa, digital platforms for real-time data collection and analysis, and the application of machine learning to HIV outcomes have increased the possibility for ‘precision public health’ to efficiently guide timely and appropriate HIV care [10,11]. Compared to traditional group-based or regression approaches, machine learning can better process complex time-varying information such as EMR data, identify non-linear or rare trends, and make accurate predictions about risk. For example, a machine learning-based hospital alert system for early sepsis detection improved patient outcomes compared to the previous scoring system manually tabulated by nurses every 12 hours [12]. Similarly, machine learning-based decision support tools could identify individuals who may benefit from ART adherence counseling, tailored SMS messages, or economic support. Such an approach could better target scarce resources to individuals most in need while minimizing cost.

Despite these potential benefits, applications of machine learning to HIV outcomes remain limited in low and middle-income country (LMIC) settings that shoulder the greatest burden of HIV [1,11]. A recent review of machine learning in the field of HIV prevention identified seven applications, five of which were in the United States, one in Denmark, and one in Eastern Africa [13]. The latter analysis used sociodemographic data collected by a population-based study in rural Kenya and Uganda to predict risk of HIV acquisition and identify potential pre-exposure prophylaxis (PrEP) candidates [14]. However, implementation of this approach may be limited in real-world clinical settings where detailed sociodemographic data are not readily available as they are in research studies.

To establish proof of concept, we applied a machine learning development-and-validation approach to routine EMR data from HIV care and treatment centers in Tanzania to identify those who are in care but at risk of disengagement, with the ultimate goal of better aligning proactive, supportive interventions to those most in need. We additionally explored whether models that also incorporated survey data from a research study could enhance the accuracy of predictions over EMR data alone.

## Methods

### Ethics statement

The Tanzania National Institute for Medical Research and the Committee for Protection of Human Subjects at the University of California, Berkeley provided ethics approval for this study. Written informed consent to participate in the study was obtained from all participants at the time of enrollment.

### Data sources and study participants

Risk of disengagement from HIV care was modeled using data about the same group of individuals from two sources: (1) EMR data from the Tanzania national HIV care and treatment center (“CTC3”) database and (2) survey data from a randomized trial of financial incentives (“Afya II”), which was conducted at 4 health facilities in Shinyanga region, Tanzania. We restricted this analysis to participants in the trial’s control arm in order to model disengagement under the current standard of care, without additional intervention. Trial methods are described elsewhere [7]; briefly, HIV-positive individuals aged 18 years or older who had initiated ART within the past 30 days were recruited during routine clinic visits in 2018. Participants provided informed consent to participate in the study and to share medical records. Sociodemographic surveys were conducted by research assistants at the time of enrollment and 6 months later. For the current analysis, participant medical records from the time of ART initiation through 24 months of follow-up were abstracted from the EMR database on April 8, 2021. This included visits to any HIV care facility in the country that was using the standard EMR (the vast majority of facilities), assuming that patients retained the same unique identification number when transferring between facilities per government protocols (but not if they restarted ART at a new facility without disclosing previous care).

### Measures

#### Outcomes

Our objective was to predict future disengagement from HIV care. We measured disengagement from care using EMR clinic attendance data, defined as ever missing an appointment for 28 or more days (a standard PEPFAR monitoring and evaluation indicator [15]) within specified intervals. This interim outcome of disengagement, which is commonly experienced by the study population, was selected for prediction because delayed and missed visits are the first steps towards eventual loss to follow-up, which we theoretically want to avert via early intervention. Specifically, our future outcomes of interest were disengagement from care during 6-month intervals (6–12 months, 12–18 months, and 18–24 months), following the standard virologic monitoring schedule in Tanzania. Separate models were developed for each 6-month interval.

#### Predictors

Predictors included in models were hypothesized to be correlated with disengagement. EMR-based characteristics included age, sex, and marital status (measured at ART initiation); measures that were regularly collected at clinic visits (typically monthly), including weight (kg), WHO clinical stage (1–4), family planning use, and antiretroviral drug (ARV) status (start, continue, substitution, or stop); and HIV viral load (copies/mL, measured every 6 months). Pregnancy status and tuberculosis treatment were also considered but were omitted due to high sparseness. Time-varying EMR variables included linear change in weight and WHO stage, and linear and quadratic changes in ARV status (S1 Text). Pre-specified predictors of interest from the research study’s baseline and 6-month surveys [7,16] included language and education (measured at baseline only), occupation, employment, head of household, household size, household socioeconomic status, cost of transportation to the facility, food insecurity [17], mental health [18], self-rated overall health, hopefulness about future health, and functional status (as defined in Table 1).

**Table 1:** Participant characteristics over time, HIV treatment initiates in Tanzania.

	6 months	12 months	18 months
	N = 178	N = 163	N = 156
**Electronic health records**			
Age (years)	36.0 (10.0)	36.8 (10.3)	37.4 (10.3)
Sex			
Male	65 (36.5%)	56 (34.4%)	55 (35.3%)
Female	113 (63.5%)	107 (65.6%)	101 (64.7%)
Marital status at ART start			
Single	34 (19.1%)	32 (19.6%)	30 (19.2%)
Married/Cohabitating	73 (41.0%)	68 (41.7%)	65 (42.0%)
Divorced/Separated	34 (19.1%)	30 (18.4%)	28 (17.9%)
Widowed	9 (5.1%)	8 (4.9%)	8 (5.1%)
*Missing*	28 (15.7%)	25 (15.3%)	25 (16.0%)
Weight (kg)	58.8 (11.3)	59.9 (11.2)	59.2 (10.8)
WHO Clinical Stage (1–4)	1.8 (0.8)	1.9 (0.9)	2.0 (0.9)
Using family planning	88 (49.4%)	67 (41.1%)	58 (37.2%)
*Missing*	12 (6.7%)	2 (1.2%)	2 (1.3%)
ARV status			
Start ARV	3 (1.7%)	0 (0%)	0 (0%)
Continue	166 (93.3%)	117 (71.8%)	145 (92.9%)
Change	8 (4.5%)	45 (27.6%)	10 (6.4%)
Stop	1 (0.6%)	1 (0.6%)	1 (0.6%)
Virally suppressed	84 (47.2%)	144 (88.3%)	148 (94.9%)
*Missing*	86 (48.3%)	8 (4.9%)	5 (3.2%)
**Survey indicators**			
Primary language is Swahili	74 (41.6%)	70 (42.9%)	65 (41.7%)
Completed primary education	114 (64.0%)	104 (63.8%)	99 (63.5%)
Farmer	39 (21.9%)	38 (23.3%)	35 (22.4%)
Worked in the past 7 days	121 (68.0%)	121 (74.2%)	116 (74.4%)
Head or joint head of household	140 (78.7%)	126 (77.3%)	119 (76.3%)
Household size ≥4 members	101 (56.7%)	94 (57.7%)	92 (59.0%)
Wealth index above median^a^	89 (50.0%)	82 (50.3%)	77 (49.4%)
Transit cost for clinic visit >2000 TZS (USD $0.86)	72 (40.4%)	68 (41.7%)	65 (41.7%)
*Missing*	16 (9.0%)	13 (8.0%)	12 (7.7%)
Food insecurity (0–27)^b^	10.4 (7.0)	9.7 (6.8)	9.6 (6.8)
Depressive symptoms^c^	52 (29.2%)	37 (22.7%)	35 (22.4%)
Anxiety symptoms^c^	31 (17.4%)	15 (9.2%)	14 (9.0%)
High self-rated overall health^d^	83 (46.6%)	92 (56.4%)	88 (56.4%)
Hopeful about health in 1 year^e^	128 (72.3%)	133 (81.6%)	127 (81.4%)
Functional limitation in the past 6 months^f^	88 (49.4%)	59 (36.2%)	57 (36.5%)

Data are n (%) or mean (SD) for the most recent observation as of each timepoint. Electronic health records were generally collected at each visit, typically monthly, except for marital status. Surveys were conducted by study staff at 0 and approximately 6 months.

^a^Wealth index created from principal components analysis of household assets and housing characteristics.

^b^Household Food Insecurity Access Scale; higher scores indicate greater food insecurity.

^c^Validated binary cutoffs of the 15-item depression subscale and 10-item anxiety subscales of the Hopkins Symptoms Checklist for Depression and Anxiety.

^d^Self-rated general health of excellent or very good vs. good, fair, or poor.

^e^Expect to be in much better health in one year vs. somewhat better, the same, or worse.

^f^Unable to work or do housework because of illness.

### Statistical analysis

#### Model development and validation

We developed supervised machine learning models with ensemble decision trees to predict risk of disengagement from HIV care. The aggregated decision tree learning approach is appropriate for this analysis that involves a smaller sample size where prediction accuracy can be substantially influenced by missing data (common in EMR data). We implemented decision tree modeling in R with the “rpart” package, which handles missingness by finding a surrogate split at each tree node wherever there is a missing value (i.e., relying on other non-missing variables that have similar prediction power).

We used a one-interval-ahead approach to build and validate future outcome models based on past predictors. For each 6-month disengagement risk interval (6–12 months, 12–18 months, and 18–24 months), we modeled observed outcomes using predictor data collected prior to the start of the interval. For example, for 6–12-month disengagement, we developed models using predictors from 0–6 months. This strategy approximated a practical application in which past visit records could be used to train the model, identify the individuals at highest risk for future disengagement, target interventions accordingly, and then continually test and improve model performance after collecting observations for the next interval. Cross-validation was performed using an 80/20 train/test split approach, whereby models were trained on data for 80% of participants and validated on data for the remaining 20% of participants, using random data splitting to ensure fairness in the algorithm [19]. The optimal tuning parameters (number of splits, number of surrogate splits, complexity of a tree, etc.) in each decision tree were selected to minimize the mean squared error evaluated by five-fold cross-validation. Lastly, to reduce the variability in a single decision tree, we adopted bagging and generated 1,000 bootstrap samples to further aggregate the prediction results across multiple trees.

For each prediction interval, we compared models using only the most current EMR data at the start of the interval such as most recent weight, WHO clinical stage, etc. (as typical in previous point-of-care risk assessment approaches) to those incorporating time-varying trends since ART initiation. Additionally, we compared models using only routinely collected EMR data to those which added survey data collected via the research study. Specifically, for each 6-month risk prediction interval (6–12 months, 12–18 months, and 18–24 months), we considered three models, using: (1) current EMR data from the last visit before the prediction interval (e.g., the 5-month visit for the 6–12 month prediction period); (2) current and time-varying EMR data from all the previous visits (e.g., 0–12 month data for 12–18 month prediction); and (3) current and time-varying EMR data and the most recent survey data (baseline or 6 months). We also conducted sensitivity analyses using only three predictors per model (targeting a shrinkage of ≤10% [20]) and compared results to assess for potential overfitting. Additionally, we conducted a sensitivity analysis using an alternative approach where models were trained using observed outcomes and predictors from the same interval (e.g., 0–6 month disengagement modeled using 0–6 month predictors) and validated on observed outcomes from the next interval (e.g., 6–12 months) for the same individuals; this approach would facilitate model development before observing future outcomes, offering practical benefit for real-time application in health facilities, although this sometimes involved temporal misalignment between predictors and outcomes during model building (i.e., predictor information were collected after disengagement occurred during the model development time period).

#### Model evaluation

We tested the performance of each model on the following 6-month validation cohorts. We evaluated the overall prediction accuracy (proportion with correctly predicted positive or negative disengagement status out of the total population) and area under the diagnostic curve (AUC). Additionally, we assessed the efficiency of each model for targeting the “highest risk” individuals in the context of limited resources where not all individuals can receive an intervention [14,21]. For set risk score thresholds corresponding to proportions of the population flagged as “high risk” (ranging from 10–50% targeted to simulate different intervention scenarios), we evaluated each model’s sensitivity (proportion correctly categorized as “high risk”—i.e., truly went on to disengage from care—out of the total population who disengaged from care in the prediction period) and positive predictive value (PPV, the proportion correctly categorized as “high risk” out of the total population categorized as high risk). Lastly, we computed the importance of each variable based on the reduction in predictive accuracy when removing the predictor of interest from each model.

## Results

### Participants

Of 184 total individuals enrolled in the control group of the trial; 1 individual who was missing all EMR records and 5 individuals who had a death recorded in the EMR during the first 6 months were excluded from this analysis, yielding a sample size of 178 participants with a total of 2,698 clinical visits over 24 months of follow-up (mean visits per participant = 17.7, SD: 6.3). Five participants with deaths recorded after 6 months (6–12 months: n = 3; 12–18 months: n = 2) were not included in models for subsequent prediction periods. In addition, participants who dropped out of care at least 6 months before later prediction periods were excluded from those models (12–18 months: n = 12; 18–24 months: n = 17). All participants completed the research study survey upon enrollment in the study, and 163 participants completed the survey again at approximately 6 months (91.6% of 178). The majority of participants were female (63.5%) with a mean age of 36 years at 6 months (Table 1).

During the first 6 months after ART initiation, 72 (40.4%) participants disengaged from HIV care (i.e., missed an appointment by at least 28 days). In ensuing 6-month periods, 44 (27.0%) participants disengaged from 6–12 months, 36 (23.1%) from 12–18 months, and 54 (30.3%) from 18–24 months. Declines in disengagement over time reflect the fact that some participants who missed visits later re-engaged in care.

### Prediction accuracy

The model using current EMR data at 6 months (from the most recent visit) predicted risk of disengagement from 6 to 12 months with 65.1% accuracy and AUC = 0.647 (95% CI: 0.577–0.745). In comparison, the EMR model using time-varying data from the first 6 months yielded 72.3% accuracy and AUC = 0.704 (95% CI: 0.530–0.878). Additionally including survey variables yielded 73.4% accuracy and AUC = 0.630 (0.409–0.861) (Table 2).

**Table 2:** Accuracy of predicting disengagement from HIV care in the next 6 months, Shinyanga, Tanzania, 2018–2021.

Time period		n/N (%) disengaged^a^		Accuracy % and AUC (95%CI) by model		
Predictors	Outcome	Training set	Testing set	Current EMR^b^	Time-varying EMR^c^	Time-varying EMR plus survey^d^
0–6 months	6–12 months	43/142 (30.3%)	16/36 (44.4%)	65.1% AUC = 0.647 (0.577–0.717)	72.3% AUC = 0.704 (0.530–0.878)	73.4% AUC = 0.630 (0.409–0.861)
0–12 months	12–18 months	37/130 (28.5%)	6/33 (18.2%)	70.8% AUC = 0.603 (0.470–0.736)	75.8% AUC = 0.722 (0.583–0.861)	77.8% AUC = 0.710 (0.476–0.944)
0–18 months	18–24 months	27/125 (21.6%)	5/31 (16.1%)	74.7% AUC = 0.640 (0.523–0.757)	77.4% AUC = 0.734 (0.586–0.882)	78.1% AUC = 0.713 (0.500–0.926)

^a^Number of individuals disengaged during the outcome period out of the total number of individuals included in the model, after removing deaths and dropouts (over 6 months since the last missed visit). Individuals were randomly split into 80% training and 20% testing sets.

^b^Using the most recent EMR value only.

^c^Using time-varying information from the whole EMR history.

^d^Using time-varying information from the whole EMR history and the most recent survey data.

Similar trends were observed for models of 12- to 18-month and 18- to 24-month risk of disengagement. For all prediction time periods, gains in accuracy resulted from models including time-varying EMR data and, to a lesser extent, sociodemographic survey data (Table 2). Results were similar in sensitivity analyses using only the three most important predictors in each model (S1 Table) and those using the past outcome development and future outcome validation approach (S2 Table).

### Prediction efficiency

**Fig 1:**
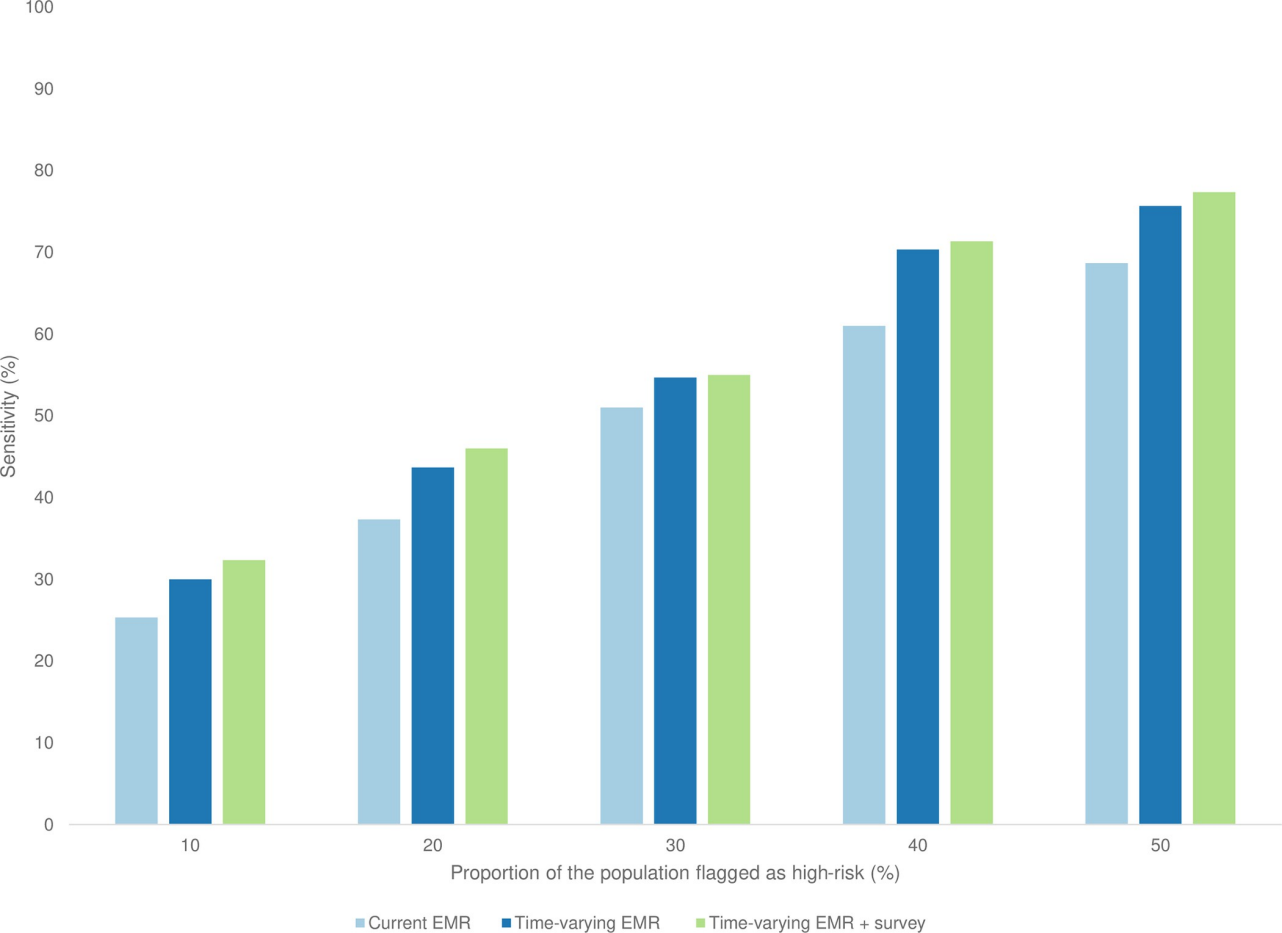
Mean model sensitivity for predicting risk of disengagement from HIV care at different risk score thresholds corresponding to the proportion of the total population flagged as “high-risk”, HIV treatment initiates in Tanzania.

When setting a threshold to identify the top 10% of individuals at highest risk of disengagement from care in the next 6 months (based on the predicted risk score generated by each model), the mean sensitivity over each time period was 25% using current EMR data only; 30% using time-varying EMR data; and 32% using time-varying EMR data along with survey indicators (Fig 1 and S3 Table). In other words, of all the individuals who truly went on to disengage from care, about 3 in 10 would be classified as “high risk” with each model (and hypothetically prioritized for intervention) if positive predictions of disengagement were limited to 10% (e.g., under a scenario where resources restrict the intervention capacity to 10% of the total population). The corresponding mean PPV (proportion of those 10% classified as high-risk who truly disengaged from care) was 68% using current EMR data only; 68% using time-varying EMR data; and 69% using time-varying EMR and survey data (S4 Table). When increasing the risk score threshold to identify the 30% highest-risk individuals, all models achieved a mean sensitivity of over 50% while also maintaining a PPV above 50% (S3 and S4 Tables).

### Variable importance

**Fig 2:**
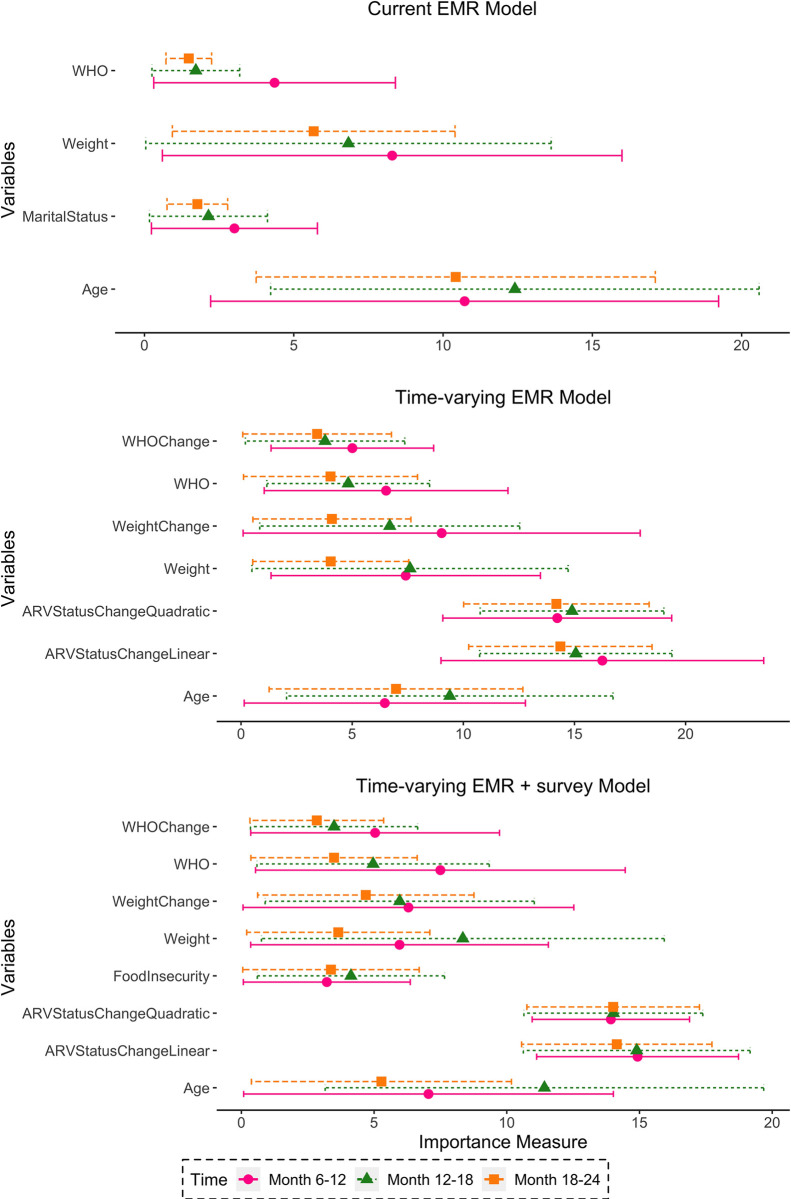
Variable importance for models predicting disengagement from HIV care, Tanzania. Plots show the top 4 most important variables for current EMR model, the top 7 for time-varying EMR model and the top 8 for time-varying EMR+ survey information models.

The strongest predictors from any model were time-varying EMR variables, including changes in ARV status, weight, and WHO clinical stage, along with age (Fig 2 and S5 Table). Food insecurity followed as an important variable in models including sociodemographic survey data.

## Discussion

Machine learning methods applied to secondary EMR data predicted future risk of disengagement from HIV care in this 2-year proof of concept study of adults who had recently initiated ART in Tanzania. Models performed especially well when incorporating changes in clinical status over time that were captured in the EMR. Additionally including survey indicators collected as part of a research study, such as food insecurity and mental health, only modestly improved accuracy; however, these data are not readily available in most clinical settings. To our knowledge, this is the first application of machine learning methods to predict retention in HIV care using routine EMR data in a low- or middle-income country.

Electronic medical records are now standard within HIV care and treatment centers in many LMIC settings including Tanzania. These readily available data present an untapped opportunity to apply predictive analytics for care optimization. Despite containing fewer variables than integrated electronic health record (EHR) systems and linked datasets in high-income countries, and possibly high levels of missingness, our results nevertheless demonstrate the potential utility of these EMR data to predict future risk of disengagement from HIV care. We implemented a novel application of machine learning to benefit from the information in the limited EMR data, using decision trees (which can appropriately handle missingness) and incorporating time-varying information into the prediction models (which was strongly predictive of disengagement status). Together, these strategies resulted in a model that could potentially be used as an early warning system for at-risk individuals in HIV care.

Our decision tree model using time-varying EMR data achieved a mean PPV of 68% for identifying the top 10% highest risk individuals as needing intervention, even without including sociodemographic indicators collected by the research study. In comparison, a machine learning analysis of complex EHR and linked geospatial data to predict dropping out of HIV care (a more rare outcome) in the United States achieved a mean PPV of 35% for identifying the 10% highest-risk individuals [22]. In Switzerland, using EHR data including electronically monitored ART adherence to predict virologic outcomes achieved a PPV of 85% [23]. Our analysis suggests that comparable results to those obtained with detailed data from high-income countries can also be achieved with current EMR data from LMICs.

As electronic record systems for HIV care continue to develop, consideration should be given to the benefits and drawbacks of adding new data collection fields. For example, EHR-based screening tools to assess social and behavioral domains are increasingly used in the United States, as part of an effort to provide patient-centered care [24–26]. In our analysis, model performance somewhat improved when including similar survey-based indicators collected by the research study, especially food insecurity (a well-documented barrier to retention in HIV care [27]). However, the strongest predictors in these models remained EMR-based measures including time-varying ARV status, WHO clinical stage, and weight. Changes in these EMR variables—or lack thereof—might capture continuity of care or changes in health status that are associated with disengagement from care. Our results suggest that these routinely collected EMR variables are among the strongest predictors of future disengagement from HIV care.

While collecting additional health and social indicators in the EMR could marginally improve risk algorithms and support individualized care, there are critical limitations in the context of already overburdened and resource-constrained settings. This extra data collection would require technological infrastructure, provider time and training to ask sensitive questions, private space within busy clinics, means and protocols for responding to surfaced needs, and data confidentiality protections. Standardized assessment of select social needs may yet be warranted for other reasons, as in the case of mental health and food insecurity, where strong arguments exist for integrated approaches to address these factors within HIV care [28,29]. However, our results show that *existing* EHR data in HIV care and treatment centers have potential for immediately use to predict future retention in care and thereby target interventions to those who could most benefit.

A limitation of this study was the relatively small sample size of participants, although two years of follow-up data per participant increased our ability to generate inference. Given the small sample size, relatively large 6-month intervals were used for predicting disengagement from care. In future work, we plan to focus on smaller intervals that would be more actionable in a clinical setting (e.g., to predict whether a patient will come to their next appointment given all information to date). We emphasize that this is a prediction exercise, rather than a causal analysis, whereby we intentionally use all of the available data to describe patterns and then extrapolate predictions into the future. In the applied clinical setting we envision, models would be developed using all of the available data from past visits and history of disengagement, and then used to predict future disengagement.

A critical strength of this study was the use of the national EMR database to capture visits from participants who transferred to different clinics after study enrollment. Still, some attended visits may have gone unobserved if transferred participants were assigned a new unique identification number (known as a “silent transfer”), or at rural clinics with unstable network connections where data syncing with the national database occurs less frequently. In addition, some attended visits may not have been captured in the EMR because of incomplete or inaccurate data entry. However, our past experience comparing abstracted paper-based clinical records (CTC2 card) to electronic records at the four study clinics has found the EMR data to be of acceptably high quality.

In conclusion, we found that machine learning methods applied to routine EMR data in Tanzania predicted future risk of disengagement from HIV care. Incorporating time-varying EMR information enhanced the prediction accuracy compared to only using point-in-time EMR data. The addition of survey data collected through a research study modestly improved the accuracy. This approach, using EMR data alone or in combination with other data sources, could potentially be used to improve the efficiency of HIV prevention and care programs by targeting supportive interventions to individuals who could most benefit.

## Supporting information

S1 Checklist TRIPOD checklist. (DOCX)

S1 Table Sensitivity analysis using only the 3 most important predictors per model. (DOCX)

S2 Table Sensitivity analysis using past outcome model development-future outcome validation. (DOCX)

S3 Table Sensitivity with respect to different predefined proportions of the population flagged as high-risk for disengagement from HIV care in the next 6 months. (DOCX)

S4 Table Positive predicted value (PPV) with respect to different predefined proportions of the population flagged as high-risk for disengagement from HIV care in the next 6 months. (DOCX)

S5 Table Variable importance by model, mean (SD, 95% CI). (DOCX)

S1 Text Description of time-varying variables. (DOCX)
