# Supplementary material for: Machine learning with routine electronic medical record data to identify people at high risk of disengagement from HIV care in Tanzania
Source: PLOS Glob Public Health. 2022 Sep 16;2(9):e0000720. doi: 10.1371/journal.pgph.0000720 (PMC10021592; doi:10.1371/journal.pgph.0000720)
Supplement: S1 Table — (DOCX) [file pgph.0000720.s002.docx]

**S1 Table. Sensitivity analysis using only the 3 most important predictors per model**

| Time period | | n/N (%) disengaged^a^ | | Accuracy % and AUC (95%CI) by model | | |
| --- | --- | --- | --- | --- | --- | --- |
| Predictors | Outcome | Training set | Testing set | Current EMR^b^ | Time-varying EMR^c^ | Time-varying EMR plus survey^d^ |
| 0-6  months | 6-12 months | 43/142 (30.3%) | 16/36  (44.4%) | 65.4%  AUC=0.649  (0.590-0.708) | 71.7%  AUC=0.699  (0.525-0.873) | 72.9%  AUC=0.701  (0.537-0.864) |
| 0-12  months | 12-18 months | 37/130 (28.5%) | 6/33  (18.2%) | 70.6%  AUC=0.621  (0.588 – 0.654) | 75.0%  AUC=0.725  (0.588-0.862) | 77.1%  AUC=0.720  (0.567-0.873) |
| 0-18  months | 18-24 months | 27/125 (21.6%) | 5/31 (16.1%) | 73.9%  AUC=0.645  (0.530-0.76) | 76.6%  AUC=0.731  (0.578-0.884) | 77.8%  AUC=0.723  (0.569-0.877) |

^a^Number of individuals disengaged during the outcome period out of the total number of individuals included in the model, after removing deaths and dropouts (over 6 months since the last missed visit). Individuals were randomly split into 80% training and 20% testing sets.

^b^Using the most recent EMR value only ( weight, age, and WHO stage)

^c^Using time-varying information from the whole EMR history (ARV status linear change, ARV status quadratic change, and weight change)

^d^Using time-varying information from the whole EMR history and the most recent survey data (ARV status linear change, ARV status quadratic change, and food insecurity)
