# Supplementary material for: Machine learning with routine electronic medical record data to identify people at high risk of disengagement from HIV care in Tanzania
Source: PLOS Glob Public Health. 2022 Sep 16;2(9):e0000720. doi: 10.1371/journal.pgph.0000720 (PMC10021592; doi:10.1371/journal.pgph.0000720)
Supplement: S2 Table — (DOCX) [file pgph.0000720.s003.docx]

**S2 Table. Sensitivity analysis using past outcome model development-future outcome validation**

| Training period | Testing  period | N^a^ | n (%) disengaged | Accuracy % and AUC (95%CI) by model | | |
| --- | --- | --- | --- | --- | --- | --- |
|  |  |  |  | Current EMR model^b^ | Time-varying EMR model^c^ | Time-varying EMR + survey model^d^ |
| 0-6 months | 6-12 months | 178 | 59 (33.1%) | 70.2%  AUC = 0.657  (0.570–0.745) | 74.2%  AUC = 0.786  (0.695–0.877) | 75.0%  AUC = 0.792 (0.706–0.878) |
| 0-12 months | 12-18 months | 163 | 43 (26.4%) | 75.5%  AUC=0.740 (0.656–0.825) | 77.5%  AUC = 0.742 (0.637–0.847) | 78.3%  AUC = 0.745 (0.617–0.873) |
| 0-18 months | 18-24 months | 156 | 32 (20.5%) | 78.9%  AUC = 0.761 (0.685–0.837) | 79.5%  AUC = 0.762 (0.680–0.843) | 80.1%  AUC = 0.783 (0.688–0.878) |

^a^Number of individuals for each model after removing deaths and dropouts (over 6 months since the last missed visit).

^b^Using the most recent EMR value only.

^c^Using time-varying information from the whole EMR history.

^d^Using time-varying information from the whole EMR history and the most recent survey data.
