# Supplementary material for: Machine learning with routine electronic medical record data to identify people at high risk of disengagement from HIV care in Tanzania
Source: PLOS Glob Public Health. 2022 Sep 16;2(9):e0000720. doi: 10.1371/journal.pgph.0000720 (PMC10021592; doi:10.1371/journal.pgph.0000720)
Supplement: S3 Table — (DOCX) [file pgph.0000720.s004.docx]

**S3 Table. Sensitivity with respect to different predefined proportions of the population flagged as high-risk for disengagement from HIV care in the next 6 months**

|  | 6-12 month disengagement | | | 12-18 month disengagement | | | 18-24 month disengagement | | |
| --- | --- | --- | --- | --- | --- | --- | --- | --- | --- |
| Limiting % of the population flagged as high-risk | Current EMR  (<6 months) | Time-varying EMR  (0-6 months) | Time-varying EMR + survey | Current EMR  (<12 months) | Time-varying  (0-12 months) EMR | Time-varying  EMR + survey | Current EMR  (<18 months) | Time-varying EMR  (0-18 months) | Time-varying EMR+ survey |
| 10 | 14 | 22 | 27 | 31 | 34 | 35 | 31 | 34 | 35 |
| 20 | 29 | 46 | 49 | 41 | 43 | 45 | 42 | 42 | 44 |
| 30 | 43 | 51 | 52 | 55 | 57 | 57 | 55 | 56 | 56 |
| 40 | 50 | 67 | 69 | 67 | 77 | 77 | 66 | 67 | 68 |
| 50 | 63 | 76 | 78 | 74 | 78 | 79 | 69 | 73 | 75 |
