# Supplementary material for: Machine learning with routine electronic medical record data to identify people at high risk of disengagement from HIV care in Tanzania
Source: PLOS Glob Public Health. 2022 Sep 16;2(9):e0000720. doi: 10.1371/journal.pgph.0000720 (PMC10021592; doi:10.1371/journal.pgph.0000720)
Supplement: S5 Table — (DOCX) [file pgph.0000720.s006.docx]

**S5 Table. Variable importance by model, mean (SD, 95% CI)**

|  | 6-12 month prediction | | | 12-18 month prediction | | | 18-24 month prediction | | |
| --- | --- | --- | --- | --- | --- | --- | --- | --- | --- |
|  | Current EMR  (<6 months) | Time-varying EMR (0-6 months) | Time-varying EMR + survey | Current EMR  (<12 months) | Time-varying EMR (0-12 months) | Time-varying  EMR + survey | Current EMR  (<18 months) | Time-varying EMR (0-18 months) | Time-varying EMR+ survey |
| Age (years) | 10.7 (4.3,  2.2–19.2) | 6.5 (3.2,  0.1–12.8) | 7.1 (3.6,  0.1–14.0) | 12.4 (4.2,  2.2-19.2) | 9.4 (3.7,  2.0–16.7) | 11.4 (4.2,  3.2–19.7) | 10.4 (3.4, 3.7–17.1) | 7.0 (2.9,  1.3–12.7) | 5.3 (2.5,  0.4–10.2) |
| Sex | 2.9 (1.0, 0.9–4.9) | 2.2 (0.9,  0.4–4.0) | 2.2 (1.0, 0.2–4.0) | 1.2 (0.5, 0.2–2.2) | 1.7 (0.8,  0.1–3.3) | 2.0 (0.8, 0.4–3.6) | 1.1 (0.5, 0.1–2.1) | 2.7 (1.3, 0.2–5.3) | 2.5 (1.1, 0.3–4.7) |
| Marital status | 3.0 (1.4,  0.2–5.8) | 4.0 (1.8,  0.5–7.5) | 2.6 (1.2,  0.2–5.0) | 2.3 (0.3,  0.2–4.3) | 3.6 (1.8,  0.1–7.1) | 3.4 (2.8,  0.3–10.5) | 1.8 (0.5,  0.8–2.8) | 3.2 (0.8,  1.6–4.8) | 2.0 (0.8,  0.4–3.6) |
| Weight (kg) | 8.3 (3.9,  0.6–16.0) | 7.4 (3.1, 1.3–13.5) | 6.0 (2.9, 0.4–11.6) | 6.8 (3.5, 0.0–13.6) | 7.6 (3.6,  0.5–14.7) | 8.3 (3.9, 0.8–15.9) | 5.7 (2.4, 0.9–10.4) | 4.0 (1.8, 0.5–7.5) | 3.7 (1.8,  0.2–7.1) |
| WHO Stage  (1-4) | 4.4 (2.1,  0.3–8.4) | 6.5 (2.3,  1.0–12.0) | 7.5 (3.6,  0.5–14.5) | 1.7 (0.7,  0.3–3.2) | 4.8 (1.9,  1.5–8.2) | 5.0 (2.2, 0.6–9.3) | 1.5 (0.4,  0.7–2.2) | 4.0 (2.0,  0.1–7.9) | 3.5 (1.6,  0.4–6.6) |
| Family planning | 2.8 (1.3,  0.3–5.3) | 2.7 (1.2,  0.3–5.1) | 3.0 (1.1, 0.8–5.2) | 1.1 (0.8, 0.7–1.5) | 1.5 (0.7,  0.1–2.9) | 1.7 (1.5, 0.2–5.4) | 1.3 (0.5, 0.3–2.3) | 1.6 (0.7, 0.2–3.0) | 1.5 (0.6,  0.2-2.7) |
| ARV Status | 1.1 (0.5, 0.1-2.1) | 4.0 (1.8, 0.5-7.5) | 2.3 (1.1, 0.1-4.5) | 1.3 (0.6, 0.1-2.5) | 3.7 (1.7, 0.3-7.1) | 3.4 (1.4, 0.8-6.2) | 0.9 (0.4, 0.1-1.6) | 2.9 (1.3, 0.3-5.4) | 2.5 (1.1, 0.3-4.7) |
| VRL | 1.0 (0.4, 0.2-1.8) | N/A | N/A | 1.3 (0.5, 0.3-2.3) | 3.3 (1.1, 1.1-5.5) | 3.0 (0.8, 1.4-4.6) | 1.0 (0.5, 0.1-1.9) | 1.2 (0.5, 0.1-2.3) | 1.2 (0.5, 0.1-2.2) |
| Weight change (kg) | N/A | 9.0 (4.6,  0.1–17.8) | 6.3 (3.2, 0.1–12.5) | N/A | 6.7 (3.0,  0.8–12.5) | 6.0 (2.6,  0.9–11.0) | N/A | 4.1 (1.8,  0.6–7.6) | 4.7 (2.1,  0.6–8.8) |
| WHO Stage change | N/A | 5.0 (1.9,  1.3–8.7) | 5.0 (2.4, 0.4–9.7) | N/A | 3.8 (1.8,  0.2–7.4) | 3.5 (1.6, 0.3–6.6) | N/A | 3.4 (1.7, 0.1–6.8) | 2.8 (1.3, 0.3–5.4) |
| ARV Status linear change^a^ | N/A | 16.3 (3.7, 9.0–23.5) | 14.9 (3.7, 11.1–18.7) | N/A | 15.1 (2.1,  10.7–19.4) | 14.9 (2.2, 10.6–19.2) | N/A | 14.4 (2.1, 10.2–18.5) | 14.1 (1.8, 10.6–17.8) |
| ARV Status quadratic change | N/A | 14.2 (2.6,  9.1–19.4) | 13.9 (1.5, 11.0–16.9) | N/A | 14.9 (2.1,  10.8–19.0) | 14.0 (1.7, 10.6–17.4) | N/A | 14.2 (2.1,  10.0–18.4) | 14.0 (1.6, 10.8-17.3) |
| Language | N/A | N/A | 3.0 (1.1, 1.0–5.0) | N/A | N/A | 2.9 (1.0, 0.9-4.9) | N/A | N/A | 3.1 (1.0, 1.1–5.1) |
| Education | N/A | N/A | 1.9 (0.9, 0.2-3.5) | N/A | N/A | 1.8 (1.4, 0.2-5.4) | N/A | N/A | 2.0 (0.9, 0.3-3.7) |
| Farmer | N/A | N/A | 2.2 (1.0, 0.2–4.1) | N/A | N/A | 2.1 (0.9, 0.3–3.9) | N/A | N/A | 2.3 (1.1, 0.2–4.4) |
| Working | N/A | N/A | 2.5 (1.0, 0.5–4.5) | N/A | N/A | 2.7 (2.4, 0.2–8.9) | N/A | N/A | 2.2 (1.0, 0.2–4.2) |
| Head of household | N/A | N/A | 1.7 (0.7, 0.2-3.2) | N/A | N/A | 2.9 (2.4, 0.3-9.1) | N/A | N/A | 2.4 (1.1, 0.2-4.7) |
| Household size | N/A | N/A | 2.0 (0.5, 1.0-3.0) | N/A | N/A | 2.2 (0.9, 0.4-4.1) | N/A | N/A | 2.1 (0.9, 0.3-3.9) |
| Wealth index | N/A | N/A | 2.1 (1.0, 0.1–4.0) | N/A | N/A | 1.9 (0.9, 0.2–3.7) | N/A | N/A | 2.2 (1.0, 0.3–4.1) |
| Transit cost | N/A | N/A | 2.4 (1.1, 0.2-4.6) | N/A | N/A | 2.0 (1.7, 0.3-6.1) | N/A | N/A | 2.4 (1.1, 0.3-4.5) |
| Food insecurity | N/A | N/A | 3.2 (1.6, 0.1–6.4) | N/A | N/A | 4.1 (1.8, 0.6–7.7) | N/A | N/A | 3.4 (1.7, 0.0–6.7) |
| Depression | N/A | N/A | 2.0 (0.9, 0.2–3.7) | N/A | N/A | 1.9 (1.6, 0.3–6.3) | N/A | N/A | 1.9 (0.8, 0.3–3.6) |
| Anxiety | N/A | N/A | 1.3 (0.5, 0.3–2.3) | N/A | N/A | 1.9 (1.8, 0.2–6.1) | N/A | N/A | 1.6 (0.7, 0.2–2.9) |
| Self-rated health | N/A | N/A | 2.0 (0.9, 0.2-3.8) | N/A | N/A | 2.2 (1.8, 0.3-6.2) | N/A | N/A | 2.2 (1.0, 0.2-4.1) |
| Functional limitation | N/A | N/A | 1.5 (0.5, 0.5-2.5) | N/A | N/A | 2.3 (2.1, 0.3-7.4) | N/A | N/A | 1.9 (0.9, 0.1-3.7) |

^a^Binary variable indicating a stop, restart, substitute, or switch to 2nd or 3rd line.
