# Supplementary material for: Machine learning with routine electronic medical record data to identify people at high risk of disengagement from HIV care in Tanzania
Source: PLOS Glob Public Health. 2022 Sep 16;2(9):e0000720. doi: 10.1371/journal.pgph.0000720 (PMC10021592; doi:10.1371/journal.pgph.0000720)
Supplement: S1 Text — (DOCX) [file pgph.0000720.s007.docx]

**S1 Text. Description of time-varying variables**

The linear change and quadratic change values for time-varying variables were obtained from separate linear regression models for each participant. The resulting coefficients for each participant were used as predictors in our models.

For example, we run the regression model $Y_{i}\sim\beta_{1}t_{i} + \beta_{2}t_{i}^{2}$ for participant *i*, where $Y$denotes ARV status (or WHO stage, weight) and $t$ denotes the number of visits. Then the coefficient $\beta_{1}$captures the linear change of ARV status with respect to time, and $\beta_{2}$captures the quadratic change. Since ARV status is a categorical variable (2 = start, 3 = continue, 4 = substitution, 5 = stop), the further $\beta_{1}$is from zero, the more changes in ARV status are observed over time. Concretely, assume patient A has ARV status = (2, 3, 3, 4, 3), then $\beta_{1}=1.59, \beta_{2}=-0.21$, and assume patient B has ARV status = (2, 3, 3, 3, 3), then $\beta_{1}=1.06, \beta_{2}=-0.14$. Comparing the two sets of time-varying coefficients implies that patient A experiences more change in ARV status over time. This simple example also demonstrates why including the quadratic term is sensible. The ARV status trajectory of patient A resembles a parabola, thus we approximate the ARV status change with an additional second-order term $\beta_{2}t^{2}.$
